# MapReduce particle filtering with exact resampling and deterministic runtime

**DOI:** 10.1186/s13634-017-0505-9

**Published:** 2017-10-18

**Authors:** Jeyarajan Thiyagalingam, Lykourgos Kekempanos, Simon Maskell

**Affiliations:** Department of Electrical Engineering and Electronics, University of Liverpool, Liverpool, L69 3GJ UK

**Keywords:** MCMC methods, Particle filters, Big data sampling, MapReduce, Resampling

## Abstract

Particle filtering is a numerical Bayesian technique that has great potential for solving sequential estimation problems involving non-linear and non-Gaussian models. Since the estimation accuracy achieved by particle filters improves as the number of particles increases, it is natural to consider as many particles as possible. MapReduce is a generic programming model that makes it possible to scale a wide variety of algorithms to Big data. However, despite the application of particle filters across many domains, little attention has been devoted to implementing particle filters using MapReduce.

In this paper, we describe an implementation of a particle filter using MapReduce. We focus on a component that what would otherwise be a bottleneck to parallel execution, the resampling component. We devise a new implementation of this component, which requires no approximations, has *O*(*N*) spatial complexity and deterministic *O*((log*N*)^2^) time complexity. Results demonstrate the utility of this new component and culminate in consideration of a particle filter with 2^24^ particles being distributed across 512 processor cores.

## Introduction

Particle filters are a Bayesian Monte-Carlo method that provide a general framework for estimation in response to an incoming stream of data. The key idea is to represent the probability density function (pdf) of the state of a system using random samples (known as particles). These samples are propagated across iterations in time in a way that capitalises on an application-specific non-linear, non-Gaussian state-space model. This state-space model describes both the dynamic evolution of the state and the relationship between the state and the measurements. The use of random samples to articulate uncertainty means that particle filters can be applied to a variety of real-world problems without any need to approximate the models used. This is in contrast to alternative techniques (e.g., the Extended Kalman filter; EKF) that approximate the models such that the uncertainty present can be approximated using a parametric probability density (a multivariate Gaussian in the case of an EKF). The result is that a particle filter typically outperforms such alternative techniques in scenarios involving pronounced departures from linear-Gaussian models. Such scenarios are widespread. This is arguably the reason why particle filters, since their inception [1], have been applied successfully in such a diverse range of contexts [2–5].

Particle filters have the appealing property that, as the number of samples increases, the ability of the samples to represent the pdf increases and the accuracy of estimates derived from the particles improves: an upper-bound on the variance of an estimate scales as $O\left (\frac {1}{N}\right)$ [6]. It is therefore natural to seek to use as many particles as possible. However, when the number of samples becomes very large, the samples will not physically fit within the memory space of a single compute node. Big data platforms have been developed to address the generic problem of which this is a special case. These platforms work by identifying abstractions of algorithms that make the potential for parallelism apparent. The platforms (and not the programmer) are then able to exploit the available computational resources to distribute the processing. One popular abstraction is MapReduce (which is described in more detail in Section 2.2). Various techniques have been developed to distribute particle filters across multiple processor cores (see Section 7 for the details), but MapReduce has not been used with particle filters extensively (that said, [7] and [8] are counter-examples we are aware of).

The resampling component is a critical component of a particle filter and non-trivial to parallelise. As will be discussed in more detail in Section 7, previous approaches to distributing the resampling step have focused on modifying the resampling process with the aim of making it more amenable to distributed implementation. One notable exception exists [9]^1^ and ensures that the output from the distributed implementation is exactly that output from a single-processor implementation while also ensuring deterministic^2^ data transfer and runtime. Such deterministic runtime is important in real-time applications (which are widespread) where the output of the particle filter is used to feed the input of another process, which needs to receive that input within a specified latency.

In this paper, we present an improved parallel implementation strategy for the resampling component, a MapReduce representation of the particle filter (including this resampling component) and instantiate the particle filter in the context of two Big data platforms. In doing so, this paper makes the following key contributions:

- We propose an improved implementation of an exact deterministic resampling algorithm that has better temporal complexity compared to the current state-of-the-art [9]. More specifically, the proposed version of the parallel algorithm has the complexity of ${\mathcal {O}}\left ((\log _{2}N)^{2}\right)$ compared to the original complexity of ${\mathcal {O}}\left ((\log _{2}N)^{3}\right)$.
- We provide two different MapReduce variants of our new algorithm that fit both with the in-memory processing and out-of-core processing models. These are the processing models used by Hadoop and Spark respectively.
- We perform detailed performance and scalability analysis of our new algorithm in comparison to both the pre-existing state-of-the-art [9] and an implementation optimised for a single-processor core. We deliberately chose an application that stresses the resampling component of the particle filter such that our analysis relates to the worst-case performance.

The remainder of this paper is organised as follows: In Section 2, we provide a brief overview of Big data processing, and the MapReduce programming model. This is then followed by a detailed description of particle filtering in Section 3. In Section 4, we describe the fundamental building blocks that are used to construct the implementations of the particle filtering algorithm, including, in Section 4.8.2, the new component of the resampling algorithm. We then describe our MapReduce-based particle filtering implementation in Section 5. We follow this section with an evaluation of our algorithms on key two MapReduce frameworks in Section 6. Section 7 highlights related work before Section 8 concludes.

## Big data processing

The focus in this paper is on the problem of using large numbers of samples within a particle filter. Big data processing frameworks (e.g., Apache projects such as: Hadoop [10], Spark [11] and Storm [12]^3^) are designed for handling large amounts of data and can therefore be applied in this context^4^. We therefore focus in this paper on using such frameworks in conjunction with parallel computational resources, such as clusters, to handle large volumes of data^5^. In this section, we discuss the use of such Big data frameworks in general and, in particular, one of the programming models that underpins such frameworks, the MapReduce programming model.

### Big data frameworks

An attractive approach for scaling the problem with data is to use Big data frameworks. More strictly, Big data frameworks go beyond the issue of data volume and address much wider issues covering augmented V’s of data, for instance *volume, velocity, variety, value* and *veracity* [13]. Big data framework-based solutions are process-centric: the programmer describes the algorithm in a way that enables the framework itself to understand (and attempt to exploit) the potential to distribute the data and processing^6^. The result of this delegation of the optimisation for speed to the framework is that, while many of today’s Big data frameworks can handle large volumes of data, none can match the runtime performance of conventional HPC systems [14].

There are a growing number of different programming models that are used to describe algorithms within Big data frameworks. These models include MapReduce [15], Stream Processing [11, 12, 16] and Query-based techniques [17, 18]. Here, we focus on one such programming model, MapReduce.

### The MapReduce programming model

**Fig. 1:**
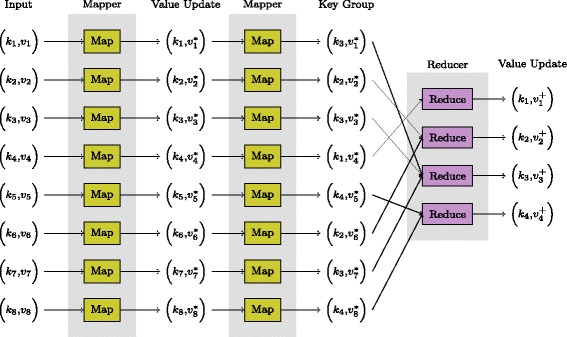
General MapReduce processing model

MapReduce is a popular programming model used in many Big data processing frameworks (and even some HPC frameworks). The key focus of the MapReduce model is on enabling the framework to distribute the processing of a large dataset by expressing algorithms in terms of *map* and *reduce* operations, via defining *mappers* and *reducers*. Mappers, when applied to each datum, output a list of *(key, value)* pairs. The framework then collates all the values associated with each key. Reducers are then applied to the list of values for each key to output a single value. Note that both the map and reduce operations are inherently parallel across all data and keys respectively^7^. To exemplify this, consider a dataset where each datum is a sentence in a Big document (e.g., Wikipedia). The problem of counting the total number of occurrences of each word in the document corpus can then be described as using the words as the key, a mapper that outputs a (non-zero) count of the number of times each word occurs in each sentence^8^ and a reducer that calculates the sum of the counts. For each word, the reducer’s output is then the sum over all sentences of the counts per sentence. Another example is shown in Fig. 1 and illustrates the ability to pass (key, value) pairs into a mapper and thereby use the output of one mapper as the input into a second mapper.

Two key frameworks that support MapReduce, albeit in slightly different ways, are Hadoop and Spark. These are now considered in turn.

#### Hadoop

MapReduce is one of the two fundamental components of Hadoop. The other is the Hadoop Distributed File System (HDFS). HDFS enables multiple computers’ disks to be accessed in much the same way as if it were a single (Big) disk. In Hadoop, the mapper and reducer generate files which are stored in HDFS, such that Hadoop implements data movement entirely via the file system.

#### Spark

The Spark framework operates using a different principle. First, at the Application Programming Interface (API) level, Spark provides a distributed data structure known as a Resilient Distributed Dataset (RDDs) [19]. MapReduce is then just one of a large number of *transformations* that (via a rich set of APIs) can be applied to RDDs. It is also important to realise that evaluations in Spark are *lazily* executed. This means, unlike conventional processing engines (e.g., Hadoop), executions never actually happen when transformations are defined. Instead, transformations are used to compose a data-flow graph and execution happens when forced through *actions* (i.e., when necessary). This delayed evaluation enables the Spark framework to optimise (and plan) the execution^9^. The result is often significant improvements in runtime performance. Another important property of RDDs is that they can reside in the memory, disk or in combination. Indeed, although Spark can make use of HDFS, the data movements in Spark are primarily via memory. Again, this can result in significant improvements in runtime performance relative to Hadoop.

## Particle filtering

We now provide a brief description of particle filtering. The reader unfamiliar with particle filtering is referred to [20]. Here, we aim to introduce notation and contextualise the discussion in the subsequent sections.

Let {**x**}_*k*=1,2,.._ be the discrete-time Markov process representing the collection of states and {**z**}_*k*=1,2,.._ be the sequence of measurements. *p*(**x**_*k*_|**x**_*k*−1_) is the state transition probability and *p*(**z**_*k*_|**x**_*k*_) is the likelihood. Recursive Bayesian filtering is the solution to the problem of using these models to process incoming data to obtain the posterior probability density function, *p*(**x**_*k*_|**z**_1:*k*_), where **z**_1:*k*_={**z**_*i*_,*i*=1,…,*k*} is the sequence of measurements up to and including time *k*. *p*(**x**_*k*_|**z**_1:*k*_) is the sufficient statistic used to calculate, for example, estimates of the current state vector.

In a particle filter, the posterior is approximated using a set of *N* random samples, where the *i*th sample is $\mathbf {x}^{i}_{k}$ and has a weight of $w_{k}^{i}$.

The weights are normalised such that $\sum _{i=1}^{N} w_{k}^{i}=1$. Estimates associated with the posterior at time *k* can then be approximated as:

1 $$ \int f(\mathbf{x}_{k})p(\mathbf{x}_{k}|\mathbf{z}_{1:k}) \approx \sum_{i=1}^{N}w_{k}^{i}f\left(\mathbf{x}_{k}^{i}\right)  $$

where *f*(.) is a function (e.g., *f*(**x**_*k*_)=**x**_*k*_ when calculating the mean). As the number of samples increases, the approximation becomes increasingly accurate. In fact, the variance of the estimate in (1) can be shown to be upper-bounded by a quantity that is proportional to $\frac {1}{N}$.

### Sequential importance sampling

Importance sampling [21] is a technique for approximating one pdf using weighted samples from another pdf. A sequential importance sampler (SIS) involves applying importance sampling to the path^10^ through the state-space, *x*_1:*k*_. The samples up to time *k* are also assumed to be generated by extending the samples of the path up to time *k*−1. This enables the weights in SIS to be derived as [22]:

2 $$ w_{k}^{i} \propto \frac{ p\left(\mathbf{z}_{k}|\mathbf{x}_{k}^{i}\right)p\left(\mathbf{x}_{k}^{i}|\mathbf{x}_{k-1}^{i}\right) } { q\left(\mathbf{x}_{k}^{i}|\mathbf{x}_{k-1}^{i}, \mathbf{z}_{k}\right) } w_{k-1}^{i}  $$

where *q*(**x**_*k*_|**x**_*k*−1_,**z**_*k*_) is the *proposal distribution* used to generate **x**_*k*_ and where

3 $$ w_{1}^{i} \propto\frac{ p\left(\mathbf{z}_{1}|\mathbf{x}_{1}^{i}\right)p\left(\mathbf{x}_{1}^{i}\right)}{q\left(\mathbf{x}_{1}^{i}\right) }  $$

where $p\left (\mathbf {x}_{1}^{i}\right)$ and $q\left (\mathbf {x}_{1}^{i}\right)$ are distributions associated with the initial state and the initial distribution of samples (both at *k*=1).

Note that, when each measurement is received, SIS involves sampling particles from *q*(**x**_*k*_|**x**_*k*−1_,**z**_*k*_) and then updating their weights using (2).

### Degeneracy problem

With the SIS algorithm, the variance of the importance weights can be proved to increase over time [22]^11^. Empirically, this results in *degeneracy*: all but one particle ends up having negligible normalised weights such that a single particle dominates the weighted average in (1). A way to quantify this effect is to calculate the effective sample size, *N*_*eff*_, introduced in [23] and estimated as follows:

4 $$ N_{eff} = \frac{1}{\sum_{i}^{N}\left(w_{k}^{i}\right)^{2}}  $$

where, since $0\leq w_{k}^{i}\leq 1$ and $\sum _{k=1}^{N} w_{k}^{i}=1$, 1≤*N*_*eff*_≤*N*.

### Sequential importance resampling

*N*_*eff*_ dropping below a threshold, *N*_*T*_, indicates that estimates are likely to be inaccurate. The key to addressing this is to introduce *resampling*. The basic idea of resampling is to eliminate samples with low importance weights and replicate samples with larger weights^12^. While there are a number of variants of the resampling algorithm, they all consist of two core stages: calculating how many copies of each sample to generate and generating that number of copies of each sample. The different resampling variants differ in terms of how they calculate the number of copies to generate. We focus here on *minimum variance resampling* (also known as *systematic* resampling) which minimises the errors inevitably introduced by the resampling process (and is discussed in more detail in Section 4.6). The use of resampling with SIS is often known as the sampling importance resampling (SIR) filter and has been at the heart of particle filters since their invention [1, 24, 25].


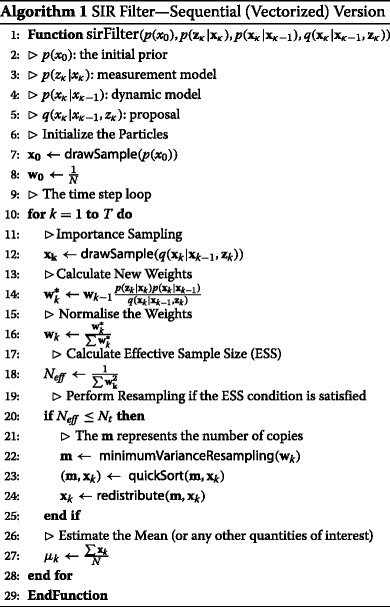


Algorithm 1 shows pseudocode for the SIR filter. Note that the algorithm is expressed in vector notation, such that each vector operation implicitly comprises at least one *for* loop and in terms of building blocks that operate on such vectors. The algorithm relies on a number of functions, which are covered in detail later in this paper. Briefly, these functions include the following:

- (a) ←drawSample(q(.)) draws samples from the supplied distribution, *q*(.);
- (m) ←minimumVarianceResampling(w) determines the number of times each particle needs to be replicated. The function takes the particles’ weights, *w*, as input.
- (m,x) ←quickSort( *m,x*) calculates the permutation that would sort vector *m* and applies this permutation to both inputs. While this sort is not necessary with a single-processor implementation, we will exploit the fact that the output has been sorted in Section 4.8.2.
- x ^′^← redistribute( *m,x*) returns the new population of particles, *x* ^′^, where *m*, as mentioned previously, defines the number of replications of each of the old population of particles, *x*.

### Parallel particle filtering

The bulk of the operations comprising the particle filter (as described in Algorithm 1) are readily parallelised. However, it is resampling (the redistribution process in particular) that complicates parallel implementation of particle filters.

The complications primarily arise because, if each of the multiple processors are considering subsets of the particles, the data transfers that the redistribution process demands are data dependent. It is therefore non-trivial to implement a particle filter in a way that the runtime is not data dependent. A similar problem has been encountered with sorting algorithms^13^. In the subsequent sections of this paper, we describe how to implement the components of the particle filter in a way that runtime is not data dependent, but deterministic.

## Parallel instantiations of the algorithmic components of particle filtering

Prior to mapping the particle filter algorithm on to a MapReduce form, it is essential to understand how the operations used by a particle filter can be implemented in a fully distributed form. While a more detailed discussion of these operations (and others) can be found in [26], we now discuss each of the operations that constitute the algorithm described in Algorithm 1. We summarise these operations and the associated complexities in Table 1, both for the fundamental building blocks and some of the algorithmic components that can be built from those components. Our focus is on implementations with a time complexity that is as fast as possible in terms of its dependence on *N*, the number of data. We discuss communication complexity for each algorithmic component by considering a simplified memory architecture where transferring a datum between two processors is considered to be a single data movement.

**Table 1:** Theoretical complexities (in terms of time, space and total data transfers per unit time) of various algorithmic components of the particle filter with *N* data and *P* processors

Section	Algorithmic component	Time	Space	Data transfers
4.1	Element-wise operations	${\mathcal {O}} (1)$	${\mathcal {O}} (N)$	${\mathcal {O}} (1)$
4.2	Rotation	${\mathcal {O}} (1)$	${\mathcal {O}} (N)$	${\mathcal {O}} (1)$
4.3	Sum/max/min	${\mathcal {O}} \left (\frac {N}{P}\log N\right)$	${\mathcal {O}} (N)$	${\mathcal {O}}(P)$
4.4	Cumulative sum	${\mathcal {O}} \left (\frac {N}{P}\log N\right)$	${\mathcal {O}} (N)$	${\mathcal {O}}(P)$
4.5	Normalising the weights	${\mathcal {O}} \left (\frac {N}{P}\log N\right)$	${\mathcal {O}} (N)$	${\mathcal {O}}{(P)}$
4.6	Minimum variance resampling	${\mathcal {O}} \left (\frac {N}{P}\log N\right)$	${\mathcal {O}} (N)$	${\mathcal {O}}(P)$
4.7	(Bitonic) sort	${\mathcal {O}} \left (\frac {N}{P}(\log N)^{2}\right)$	${\mathcal {O}}{(N)}$	${\mathcal {O}}(P)$
4.8.1	Redistribution from [9]	${\mathcal {O}} \left (\frac {N}{P}(\log N)^{3}\right)$	${\mathcal {O}} (N)$	${\mathcal {O}} (P)$
4.8.2	Improved redistribution	${\mathcal {O}} \left (\frac {N}{P}(\log N)^{2}\right)$	${\mathcal {O}} (N)$	${\mathcal {O}} (P)$
6.1.1	Naïve redistribution	${\mathcal {O}} (N)$	${\mathcal {O}} (N)$	${\mathcal {O}} (1)$

### Element-wise operations

Perhaps the simplest type of operation to implement in parallel involves applying an element-wise operation^14^. Given a function *f* and a vector **v**, the element-wise operation *f*↦**v** applies the function *f* on every element of the vector such that

$$f\mapsto\mathbf{v} = \left[f(v_{1}), f(v_{2}), \ldots, f(v_{N})\right] $$

In our case, normalizing the weights is an example of an element-wise operation. Another example is a vector of *if* operations, *Vif*(**a**,**b**,**c**) where the *i*th element in the output is *b*_*i*_ if *a*_*i*_ is true and *c*_*i*_ otherwise.

It should be evident that operations that involve two inputs and a single output (e.g., element-wise sum or difference) are similarly easy to implement in parallel and involves no data movement between processors.

### Rotation

Another operation that we will use involves rotating (with wrapping (i.e., cyclic shift) or without) the elements of a vector by a given distance, *δ*, such that if the input is **a** and the output is **b**, after the rotation, we have *b*(mod (*i*+*δ*,*N*))=*a*(*i*) where mod(*x*,*y*) is *x* modulus *y*. Once again, this algorithmic component is readily parallelised with no data movements between processors.

We will also use partial rotations such that we have a vector of distances, *Δ*, and not a single ‘global’ distance, *δ*. This vector, *Δ*, has *N* ^′^<*N* elements where *N* ^′^ is a power of two. The rotations are then implemented locally to each set of $M=\frac {N}{N'}$ elements. For example, if the *j*th element of *Δ* is *δ*_*j*_ then *b*((*j*−1)×*M*+mod(*i*+*δ*_*j*_,*M*))=*a*((*j*−1)×*M*+*i*) for 1≤*i*≤*M*.

### Sum, max and other commutative operations

**Fig. 2:**
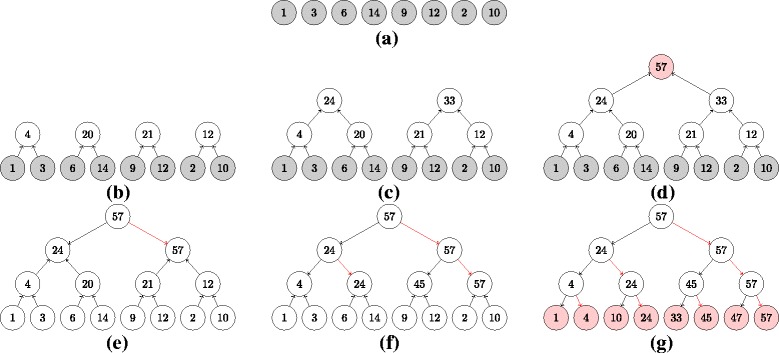
Example of cumulative sum for *N*=8 numbers. Subfigures **a**–**d** describe the sum computation, while the remaining balanced binary trees shown in subfigures **e**–**g** describe how the backward pass culminates in calculation of the cumulative sum of the given sequence

To calculate a sum of a vector of numbers, we can use an ‘adder tree’. The numbers are associated with the leaves of the tree. By recursing up the tree, the sum of the pairs of numbers can be calculated (in parallel across all pairs). The sum of all pairs of pairs of numbers can then be calculated (in parallel across all pairs of pairs). This is exemplified in Fig. 2(a–c). This process can repeat until we reach the root node of the tree and calculate the sum of all the numbers by summing the sum of the two halves of the data. See Fig. 2 (d).

In fact, as has been known since the development of the infamous Array Programming Language (APL) [27], this same approach can be used for any binary operation, ⊕, that is commutative such that

5 $$ ((a\oplus b)\oplus c)\oplus d=(a\oplus b)\oplus(c\oplus d)  $$

Relevant examples of operations which can be calculated in this way include the sum but also the maximum (and minimum) and first non-zero element of a set of numbers (which we will denote First(.) in, for example, algorithms ?? and ??). For such operations, with *N* processors processing *N* data and a binary tree, the time complexity is the depth of the tree, i.e., log2*N*. Note that, near the bottom of the tree, the total communication required is proportional to the number of processors.

As should be evident, an upside-down version of the same tree can be used to implement an Expand(*a*) operation, which involves making all elements of a vector equal to the single value of *a*.

### Cumulative sum

While the ability to use a tree to calculate a sum efficiently is well known, the ability to use a closely related approach to calculate a cumulative sum^15^ efficiently appears to be less well known by researchers working on particle filters. Of course, a naïve implementation involves computing the cumulative sum by simply adding each element of the input to the previous element of the output. Such an approach would have a runtime of *N*. However, a more efficient approach has existed since the development of APL if not for longer^16^.

To ensure that the reader has some intuition as to how this could be possible, the key idea is to exploit the partial sums that are calculated in an adder tree and to express each element of the cumulative sum as a sum of these (efficiently calculated) partial sums. The process that exploits this insight then involves a second tree in which the values at every level are propagated to the level below, replacing the values that were calculated in the adder tree. More specifically, in the downward propagation, the value at each parent node is propagated to its right child and to its left child. The new value for the left child is the difference between the value at the parent node and the value at the right child node (as calculated in the adder tree). The new value for the right child is just the same value as the parent node. See Fig. 2(e–g) for an example.

With this forward and backward pass of the tree, we can obtain the cumulative sum in 2 log2*N* steps.

### Normalising the weights

Normalising the weights is an example of an operation that can be implemented using the building blocks described to this point. The sum is calculated using an adder tree (as described in Section 4.3), distributed to all the data (as also described in Section 4.3) and an element-wise divide (see Section 4.1) used to calculate the normalised weights.

### Minimum variance resampling

As explained in Section 3.3, resampling involves determining the number of copies of each particle that are needed. We specifically describe minimum variance resampling, for which the number of copies of the *i*th particle is

6 $$ m_{i} = \lfloor C_{i}\times N\rfloor-\lceil C_{i-1}\times N\rceil+1  $$

where ⌈*x*⌉ and ⌊*x*⌋ are respectively the ceiling^17^ and the floor^18^ of *x*, where

7 $$ C_{i} = \sum_{j=1}^{i} w_{i}+\epsilon  $$

is the cumulative sum and where $\epsilon \sim \left [0,\frac {1}{N}\right ]$ and *C*_0_=0.

(6) uses only element-wise operations (as described in Section 4.1) and a rotation (by a single element and as described in Section 4.2). (7) involves a cumulative sum (as described in Section 4.4) and an addition (as described in Section 4.1). Thus, the building blocks described to this point can be used to implement (6) and (7).

### Sorting

Quicksort [28] is well known and has an average time complexity of ${\mathcal {O}} (N\log _{2} N)$. However, we focus on the bitonic sort algorithm [29], which has a time complexity of ${\mathcal {O}} \left (\frac {N}{P}\left (\log _{2} N\right)^{2}\right)$ and a spatial complexity of ${\mathcal {O}} (N)$. The number of data movements at each iteration is *P*. The main reason for this choice is that we want to guarantee the time taken to perform sorting. While it is possible to parallelise quicksort, the ability to do so is data dependent. In contrast, bitonic sort has deterministic time complexity (with a balanced load across (up to) *N* processors).

At the fundamental level, a *bitonic sequence* forms the basis for the bitonic sort. A sequence **a**=[*a*_1_,*a*_2_,…,*a*_*N*_] is a bitonic sequence if *a*_1_≤*a*_2_≤…≤*a*_*k*_≥…≥*a*_*N*_ for some *k*, 1≤*k*≤*N* or if this condition holds for any rotation of **a**.

To try to provide some intuition as to how the algorithm works, note that at a certain point it the algorithm, we have *N* data in a bitonic sequence. The first ‘half’ of the data are sorted in an ascending order and the second half are sorted in a descending order^19^. Consider the *i*th element in the first half and the *i*th element in the second half. There are $\frac {N}{2}-1$ data between these two elements. They must all be larger than the smallest of the two elements which the data are between. There must therefore be at least $\frac {N}{2}$ data that are larger than the smallest of the two elements. This smallest element must therefore be one of the lowest $\frac {N}{2}$ data (it cannot be one of the largest $\frac {N}{2}$ data if there are at least $\frac {N}{2}$ data larger than it). An upside-down version of the same argument makes clear that the largest of these two elements must be one of the largest $\frac {N}{2}$ data. Finally, it also transpires that after this operation, the first $\frac {N}{2}$ data are a bitonic sequence and the second $\frac {N}{2}$ data are a bitonic sequence. Thus, given a bitonic sequence, by comparing all pairs of data that are a distance of $\frac {N}{2}$ apart and swapping the points if needed, we can ensure all the larger elements are in the first $\frac {N}{2}$ data, which forms a bitonic sequence, and all the smaller elements are in the second $\frac {N}{2}$ data, which also forms a bitonic sequence. We can then apply the same comparison structure on each of the two bitonic (smaller) sequences. This process can be applied recursively until pairs of points are compared and the data are sorted.

This process is known as the ‘bitonic merge’ and requires ${\mathcal {O}} (\log _{2} N)$ steps (with ${\mathcal {O}} (N)$ spatial complexity) to convert a bitonic sequence into a sorted sequence. To generate the bitonic sequence needed from the arbitrary input data^20^, we apply bitonic sort to put the first $\frac {N}{2}$ input data into an ascending order and apply bitonic sort again to put the second $\frac {N}{2}$ input data into a descending order. Analysis of this recursive use of bitonic sort gives rise to bitonic sort requiring $\frac {n^{2} - n}{2}$ iterations where *n*= log2*N* and, at every step, the algorithm performs $\frac {N}{2}$ comparisons. Each comparison involves comparing two data and swapping them according to a criterion that is defined by the position of the comparison in the network (and can be implemented using the building blocks described in Sections 4.1 and 4.2).

**Fig. 3:**
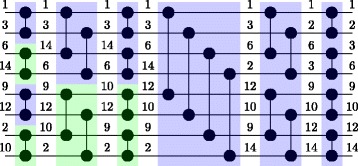
Example of bitonic sort using eight numbers. Each horizontal wire corresponds to a core. The blue color denotes that the larger value will be stored at the lower wire after the comparison, while the green color the opposite

An example of bitonic sort with eight numbers is provided in Fig. 3.

### Redistribution

#### Original version from [9]

The redistribution algorithm takes two inputs, the old population of particles **x** and the number of copies **m**, and produces the new population of particles, **x** ^∗^, as the output.

**Fig. 4:**
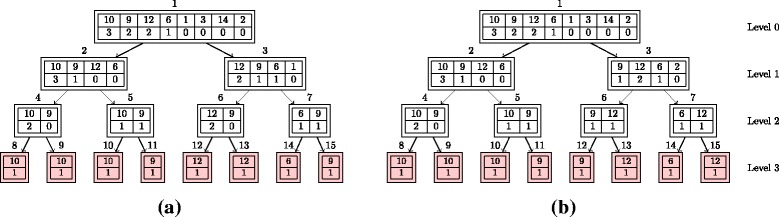
An example of the redistribution for **x**=[10,9,12,6,1,3,14,2] and **m**=[4,2,1,1,0,0,0,0] using the original and improved (new) redistribute. The original redistribution always sorts the number of copies vector (bottom vector) in a descending order, while this is not required in the new redistribution (e.g., see node no. 3). **a** Original. **b** New

In [9], a divide-and-conquer algorithm was described for implementing the redistribute. The procedure involves sorting the particles in decreasing order of the number of copies. With *N* data, the sum of the elements of **m** must be *N*. The approach is then to divide the data into two smaller datasets, each of which has $\frac {N}{2}$ elements and is such that the corresponding elements of **m** are sorted and sum to $\frac {N}{2}$. This can be achieved by finding the *pivot*, which we define as the leftmost element in **m** for which the associated value of the cumulative sum is $\frac {N}{2}$ or greater. In general, the pivot needs to be split into two constituent parts such that the two smaller datasets can both sum to $\frac {N}{2}$. We refer to these two parts as the left pivot and right pivot. The data to the left of the pivot and including the left pivot can be used to produce one of the two smaller datasets. The right pivot and the data to the right of the pivot can be used to produce the other of the two smaller datasets. Both smaller datasets are then sorted^21^ such that they are in decreasing order of **m**. Note that there is a special case that occurs when the value of the right pivot is zero: the rotation needed is one less than otherwise in this case. It can be intuitive to think of this procedure as operating on a tree. Applying the procedure recursively down the tree, until the leaf nodes are encountered, results in the redistribute completing. See Fig. 4 for an illustrative example of this procedure.

A few points are worth highlighting: the operation of the algorithm is not dependent on **m** and not dependent on the distribution of the weights; sort can (somewhat counter intuitively and seemingly unnecessarily) change the order of numbers in a list when elements of the list are not unique; if no copies of a particle are to be generated, the identity of the corresponding particle is irrelevant to the eventual output of the algorithm.

The procedure can be described using element-wise operations (see Section 4.1), sum (see Section 4.3), cumulative sum (see Section 4.4), rotations (see Section 4.2) and sort (see Section 4.7). Algorithm 2 provides a description of this algorithm. Note that the description makes use of three functions (*LeftHalf*(.), *RightHalf*(.) and *Combine*(.)) which are included to aid exposition (and actually have zero computational cost). Also note that the implementation is described in a way that involves recursion. It is possible to ‘unwrap’ the recursive implementation such that all operations (at all stages in the tree) are implemented on datasets of the same size (a size of *N*). Doing so is conceptually straightforward though the bookkeeping required is non-trivial.


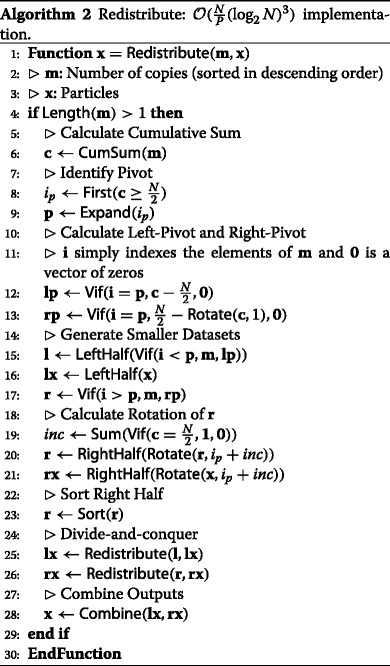


The time complexity of this redistribution algorithm ${\mathcal {O}}\left (\frac {N}{P}(\log _{2} N)^{3}\right)$ in parallel with *N* processors since a (bitonic) sort (with complexity of ${\mathcal {O}}\left (\frac {N}{P}(\log _{2} N)^{2}\right)$) is used at each stage in the divide-and-conquer. Note that this contradicts the (erroneous) claim in [9] that the time complexity of this algorithm is ${\mathcal {O}}\left (\frac {N}{P}(\log _{2} N)^{2}\right)$. The communication complexity is (again) *P*.

#### Improved redistribution

The redistribution algorithm described in Section 4.8.1 is a divide-and-conquer algorithm that ensures that, at each node in the tree, **m** sums to its length, *N*, and is sorted. The sorting is sufficient to ensure that rotation can be used to replace some of the (rightmost) zeros with the (rightmost) non-zero elements of **m** that sum to $\frac {N}{2}$.

Here we exploit the observation that it is possible to define an alternative divide-and-conquer strategy. More specifically, we ensure that, at each node in the tree, **m** sums to its length, *N*, and has all its non-zero values to the left of all values that are zero. Since such a sequence only has trailing zeros, we call such a sequence an All-Trailing-Zeros (ATZ) sequence^22^. While a sort is sufficient to generate an ATZ sequence, it is easier, as we will demonstrate shortly, to generate an ATZ sequence than it is to generate a sorted sequence.

The new algorithm, at each node in the tree, starts with **m**, which sums to its length, *N*, and is an ATZ sequence. To proceed, as previously, we find the pivot (as defined in Section 4.8.1). As previously, the data to the left of the pivot and the left pivot can be used to produce one of the two smaller datasets. However, in contrast to the approach described in Section 4.8.1, we can simply use the right pivot and the data to the right of the pivot to generate the second smaller dataset (without any need for sort). Both these smaller datasets then sum to $\frac {N}{2}$ and are ATZ sequences. Note that, as with the approach described in Section 4.8.1, there is a special case that occurs when the value of the right pivot is zero.

To initiate the algorithm, we need to generate an ATZ sequence. To achieve this, we propose to use (bitonic) sort (once). After this initial sort, the procedure can be described using element-wise operations (see Section 4.1), sum (see Section 4.3), cumulative sum (see Section 4.4) and rotations (see Section 4.2). We emphasise that there is no need for a sort after the initial generation of an ATZ sequence. As a result, while the algorithm described in Section 4.8.1 has time complexity of ${\mathcal {O}}\left (\frac {N}{P}(\log _{2} N)^{3}\right)$, the algorithm described in this section has time complexity of ${\mathcal {O}}\left (\frac {N}{P}(\log _{2} N)^{2}\right)$. Notice that the number of data movements is still *P*. To aid understanding, algorithm 3 provides a description of this algorithm. Note the very strong similarity to algorithm 2 and that, once again, it is possible to ‘unwrap’ the recursive implementation albeit with some non-trivial bookkeeping.


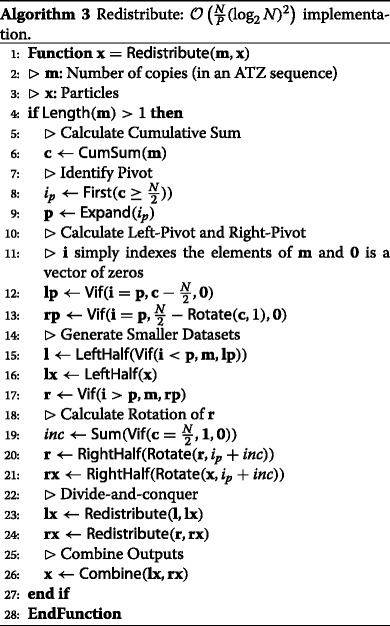


## Mapping particle filtering into MapReduce

The descriptions provided in the Section 4 describe distributed operations that can manipulate vectors (albeit after some unwrapping of the recursive descriptions).

As discussed in the Section 2.2, the fundamental notion of MapReduce is the processing of (key, value) pairs. In the context of particle filtering, none of the properties of the particles (weight or state) qualifies to be a key. However, we can give each particle a unique index and use this index as the key, such that we think of the particles as being a set {*i*,*x*_*i*_,*w*_*i*_} where *i*∈{1,…,*N*} and, as previously mentioned, where *N* is the number of particles, *x*_*i*_ is the state and *w*_*i*_ is the corresponding weight of the *i*th particle.

## Evaluation

We performed extensive evaluation of our algorithm on two different systems. We provide the details of these systems in Table 2. The evaluation process included the algorithms outlined in the Section 4 on the two key frameworks that support MapReduce and which were mentioned in Section 2: Hadoop and Spark. We used the standard estimation problem (involving a scalar state and a computationally inexpensive proposal, likelihood and dynamic model) that is widely used in the particle filtering community [20]. We perceive this scenario emphasises the need for efficient resampling, where, as is often the case, the likelihood, dynamics and proposal were computationally demanding and the relative merits of different resampling schemes would be less apparent. Our evaluation focused on specific aspects of the implementation, which are described as follows:

1. We start, in Section 6.1, by providing evidence that, in contrast to a naïve implementation, the particle filter we have developed can exploit multi-core architectures while having deterministic runtime.

**Table 2:** Details of the experimental platform used for evaluation

Details	Single node system	Multi-node system
Name	Platform 1	Platform 2
Number of nodes	1	28
Hardware cores	16	512
Operating system	Linux	IBM Unix
Primary memory	16 GB	384 GB
Spark version	1.6.2	1.4.1
Hadoop version	2.7.2	2.7.1
2. In Section 6.2, as a precursor to a detailed evaluation and analysis, we analyse the overall profile of the particle filtering algorithm for implementations on a single core, using Hadoop and using Spark.
3. Then, in Section 6.3, for both the Spark and Hadoop implementations, we compare the performance of our new algorithms relative to a single mapper and a single reducer. In doing so, we not only compare the overall performance, but we also compare the fundamental building blocks of the particle filtering algorithm. This section provides a thorough understanding of these algorithms’ performance on two key frameworks that support MapReduce.
4. Given that the Spark implementation (unsurprisingly) outperforms the Hadoop implementation, we then focus on the Spark implementation. In Section 6.4, we then compare the two versions of the redistribution algorithm described in Sections 4.8.1 and 4.8.2 as a function of the numbers of particles and cores. The intent is that this detailed comparison provides insight into the performance that is achievable using the original and proposed variants of the redistribution algorithm.
5. Finally, in Section 6.5, we perform a detailed analysis on the speedup and scalability of the redistribution and the overall particle filter.

In performing these evaluations, a basic parameter that we found useful in assessing the algorithmic performance is the capability to process large amount of data, which directly translates to the number particles that can be processed per unit time, the number of particles processed per second (PPS).

### Worst-case runtime performance

#### Baseline redistribution algorithm

We will compare performance against a naïve baseline implementation of the redistribution component. This implementation involves calculation (in parallel) of a cumulative sum of the number of copies. Once this cumulative sum is calculated (and each element of the sum communicated to be processed along with its neighbour), for each particle in the old population, we know the first and last indices of particles in the new population that will be copies of this particle in the old population. Then, by performing a loop across the particles in the old population, we can populate the new generation of particles (Algorithm 4). In MapReduce, a map function is used for the outer for loop and within each map, we iterate as many times as needed according to the number of copies elements.


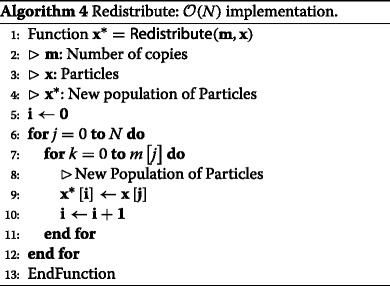


Note that this algorithm, when running across multiple cores, can be expected to have a runtime complexity that is dependent on the data. To help make this clear, consider the worst case where the redistribution involves making *N* copies of the *i*th particle (and zero copies of all other particles). In this case, only one core will actually be populating the new generation of particles.

#### Runtime performance and variability

**Fig. 5:**
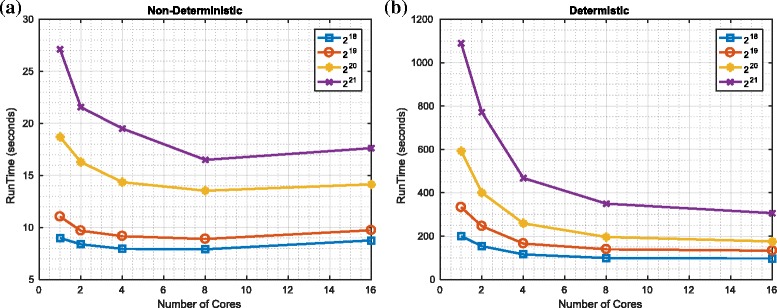
Worst-case performance of redistribution: platform 1. **a** Naïve implementation. **b** Proposed approach

**Fig. 6:**
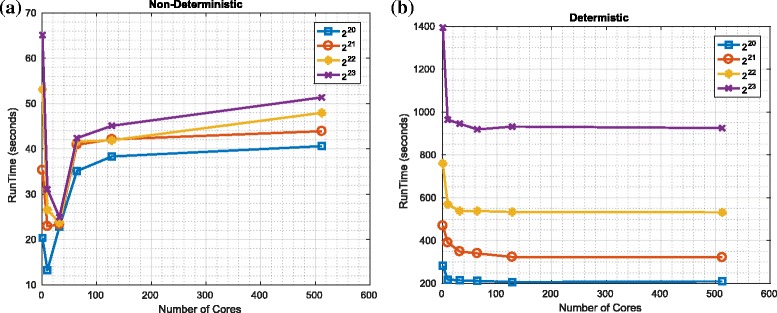
Worst-case performance of redistribution: platform 2. **a** Naïve implementation. **b** Proposed approach

We investigated the worst-case performance of such a naïve parallel implementation of the redistribution component and compared with our proposed implementation (using a Spark implementation). The results are shown in Figs. 5 and 6 for the worst case (where the new population of particles are all copies of a single member of the old population). It should be evident that as the number of cores increases, the runtime of the proposed (almost) never increases^23^. In contrast, while the runtime of the naïve implementation initially decreases as the number of cores is increased, it then increases (i.e., such that it is faster in absolute terms to use 8 not 16 cores with platform 1 and such that it is faster to use less than 50 cores not 512 cores with platform 2). The reason for the decrease is that the MapReduce framework can use the extra cores to more rapidly process the (many) zeros in the vector describing the number of copies. The reason for the subsequent increase in processing time is that the additional overhead of having multiple cores becomes increasingly significant if only one of the cores is doing the vast majority of the processing.

It should also be evident that the absolute runtime (on these platforms and with our current Spark implementation) of the deterministic and non-deterministic variants differ significantly such that the naïve implementation can be approximately 20 times faster (in the contexts of both platforms). This is disappointing and does motivate future work to refine our (initial) implementation. However, we perceive that there are applications where a slower but deterministic runtime is preferable to a faster but data-dependent runtime. In the contexts of such applications, particularly given the scope to improve the implementation, we perceive our algorithm (if not our current implementation) has utility.

**Fig. 7:**
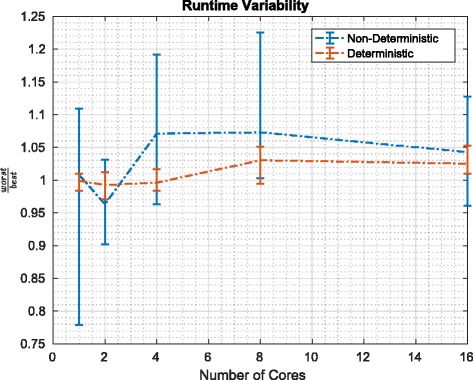
Ratio of average (and minimum and maximum) runtimes for worst-case and best-case scenarios using the deterministic and naïve redistribute

To assess the variation in runtime that we experience empirically when considering different distributions of the weights, we compared the performance in the context of the worst-case scenario with the performance in the best-case scenario^24^. Figure 7 describes the average runtime (as well as the minimum and maximum runtimes) over five runs. It is immediately clear that the fluctuations between the runs are smaller in the context of the deterministic than in the context of the naive non-deterministic algorithm. What is perhaps less clear, but still discernable, is that the average runtime for the deterministic redistribute is impacted less by moving from the worst-case to the best-case scenario than the naive non-deterministic redistribute. We believe that this modest difference points to the runtime being dominated by things other than the algorithmic choice: for example, MapReduce’s overheads (which are common to both algorithms’ implementations).

### Overall profile

**Fig. 8:**
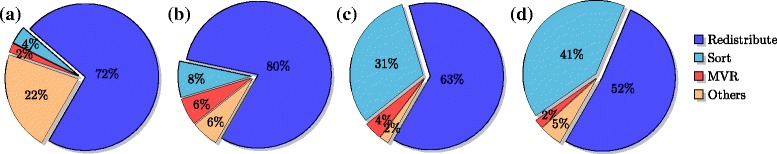
Overall runtime profile of the particle filtering algorithm for the following implementations: **a** Sequential. **b** Hadoop. **c** Spark with 2^17^ particles. **d** Spark with 2^20^ particles

We now compare performance of three implementations of a particle filter: a sequential implementation (in Java and using quicksort in place of bitonic sort), an implementation in Hadoop and an implementation in Spark. All implementations involve a single core and platform 1. Figure 8 shows the proportion of the runtime that is associated with redistribution, sort, minimum variance resampling (MVR) and the remaining components (e.g., sum, cumulative sum, diff, scaling).

As can be observed from Fig. 8, the majority of the time taken is devoted to the redistribution component. Note that, for the Spark implementation, a significant fraction of the remaining time is spent on the sorting component and the fraction of time devoted to redistribution and sorting increases as the number of particles is increased.

### Comparison of Hadoop and Spark

We next investigate how the choice of middleware impacts performance in the context of the components of the algorithm and in the context of the entire particle filter algorithm. All implementations involve a single core and platform 1.

#### Sum and cumulative sum

**Fig. 9:**
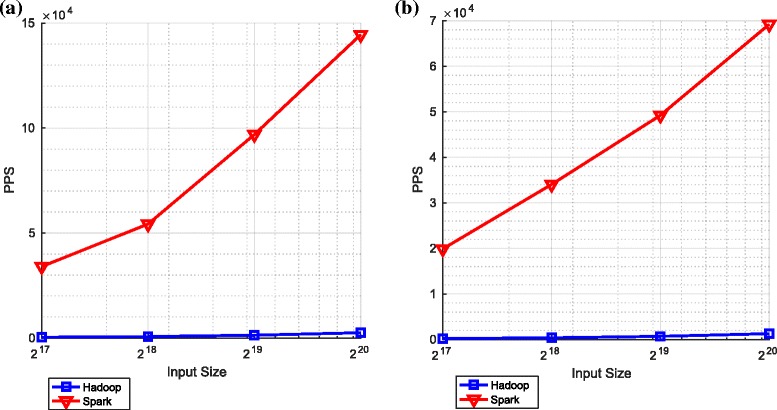
Summation and cumulative summation on Spark and Hadoop. **a** Summation. **b** Cummulative summation

Figure 9 shows the comparative performance of the sum and cumulative sum components in these two key frameworks.

With respect to the number of particles processed per second (PPS), the performance using Spark is far superior to that achieved using Hadoop. This stems from the issues discussed in Section 2: Spark uses RDDs to makes use of memory (and lazy evaluation) whereas Hadoop only uses the file system (HDFS) to transfer data from the output of one operation to the input of the next.

It is apparent in both frameworks (and particularly apparent in the context of Spark) that, as the number of particles increases, the number of particle processed per second also increases. This is because with more particles, the overheads associated with setting up (and tearing down) the mappers and reducers are increasingly offset by the parallel operations that make use of the mappers and reducers. The limited extent to which this effect is observed in the context of Hadoop highlights that the overheads associated with opening files in HDFS are significant.

Since, as explained in Section 4, calculating a summation involves one adder tree and cumulative sum involves two such trees, we should expect the number of particles per second for the cumulative sum to be approximately half that for the summation. A comparison of the two graphs in Fig. 9 makes clear that this is indeed (approximately) the case for both frameworks and for all input sizes.

#### Bitonic sort and minimum variance resampling

**Fig. 10:**
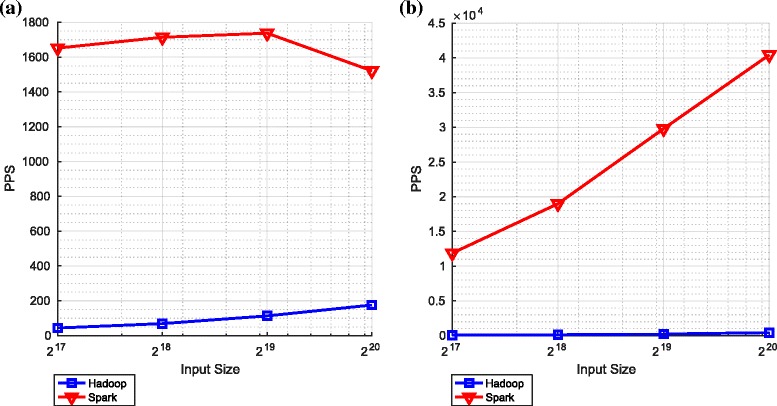
Bitonic sort and minimum variance resampling on Spark and Hadoop. **a** Bitonic sort. **b** Minimum variance resampling

Figure 10 shows the performance for two independent components, *bitonic sort* and *minimum variance resampling*. The performance of *minimum variance resampling* is relatively close to the performance of the cumulative sum (see Fig. 9). This is expected since, as explained in Section 4, *minimum variance resampling* includes a cumulative sum.

Once again and for the same reasons as discussed in Section 6.3.1, we notice the same difference in performance between the Spark and Hadoop implementations. As one might expect and as before, for *minimum variance resampling*, the number of particles per second increases with the number of particles. However, it is noteworthy that, for bitonic sort with Spark, the number of particles per second decreases for large numbers of particles. On investigating this in some detail, we observed that the *lineages* used to facilitate the lazy evaluation in Spark^25^ become very large with large numbers of particles. This appears to cause Spark to become less efficient when the number of particles becomes large.

#### Redistribution and overall performance

**Fig. 11:**
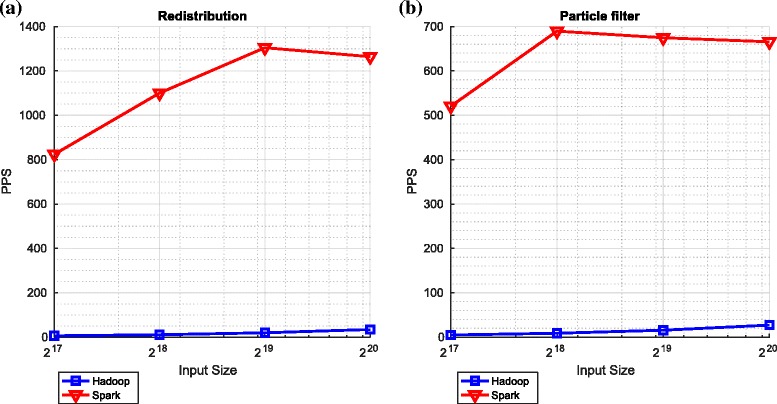
Redistribution and the overall particle filtering on Spark and Hadoop. **a** Redistribution. **b** Overall particle filtering

Finally, Fig. 11 shows the comparative performance of the redistribution algorithm (as described in Algorithm 3) and the overall particle filtering algorithm.

Once again, we notice the same differences between Hadoop and Spark. In the context of the overall particle filter and for the largest number of particles considered, these differences are manifest in Spark, relative to Hadoop, offering a considerable speedup (approximately 25-fold^26^).

The overall performance of the particle filtering algorithm, when implemented in Spark, decreases for large numbers of particles. Again, on investigation, this appears to be caused by large lineages associated with the large number of particles. Finally, we note that the bitonic sort and redistribution components appear to be limiting the number of particles per second that can be processed by the overall particle filtering algorithm.

### Impact of using multiple cores

We now focus on the Spark implementation (with platform 1) and compare the performance of the two variants of the redistribution component in isolation and in the context of the overall performance of a particle filter. More specifically, we investigate how performance scales with the number of cores and the number of particles.

#### Redistribution component in isolation

**Fig. 12:**
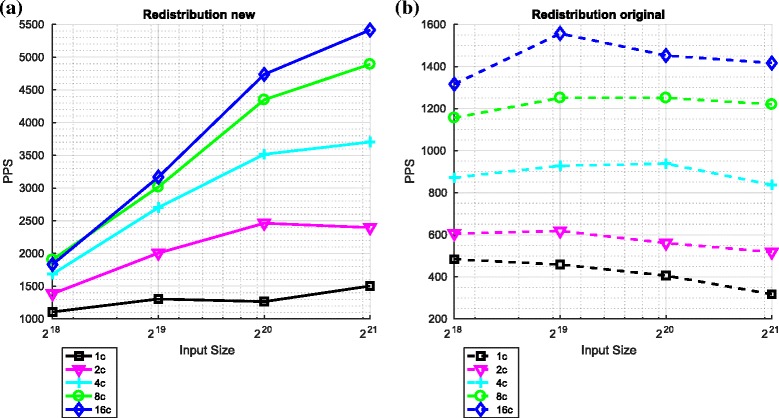
Performance of the two variants of the redistribution component (using Spark). **a** Redistribution ${\mathcal {O}} \big (\frac {N}{P}({log}_{2}N)^{2}\big)$. **b** Redistribution ${\mathcal {O}} \big (\frac {N}{P}({log}_{2}N)^{3}\big)$

Figure 12 compares the performance of the two versions of the redistribution component as a function of the number of particles and number of cores.

On a core-to-core basis, the ${\mathcal {O}} \left (\left (\log _{2} N\right)^{2}\right)$ redistribution component outperforms the ${\mathcal {O}} \left (\left (\log _{2} N\right)^{3}\right)$ component across all numbers of particles by a margin of up to a factor of approximately 4 (for 16 cores).

For all numbers of particles, increasing the number of cores improves performance for both variants of the redistribution component. However, in the context of both variants, the improvement in performance when considered as a ratio is less than the ratio of the number of cores.

In the context of the ${\mathcal {O}} \left (\frac {N}{P}\left (\log _{2} N\right)^{3}\right)$ variant, increasing the number of particles for a fixed number of cores can significantly reduce the number of particles processed per second. This is not the case for the ${\mathcal {O}}\left (\frac {N}{P}\left (\log _{2} N\right)^{2}\right)$ variant.

For the ${\mathcal {O}} \left (\frac {N}{P}\left (\log _{2} N\right)^{2}\right)$ variant, increasing the number of particles while keeping the number of cores constant improves the number of particle processed per second. However, in the context of the ${\mathcal {O}}\left (\frac {N}{P}\left (\log _{2} N\right)^{3}\right)$ variant, increasing the number of particles for a fixed number of cores can reduce the number of particles processed per second.

#### Resulting overall particle filter performance

**Fig. 13:**
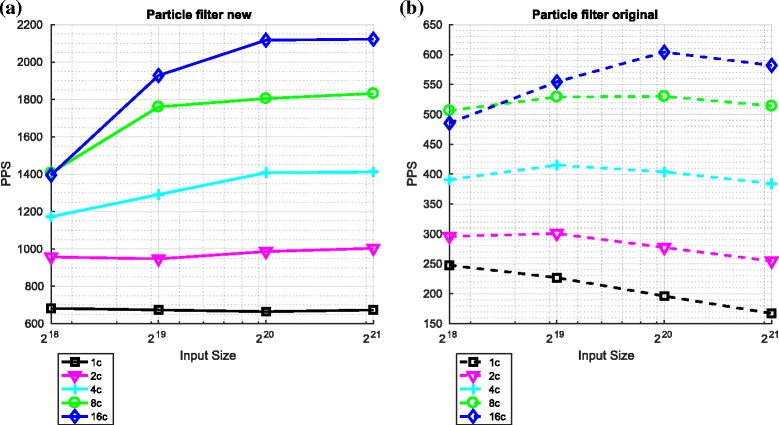
Performance of the overall particle filter using the two variants of the redistribution component. **a** Particle filter ${\mathcal {O}} \big (\frac {N}{P}({log}_{2}N)^{2}\big)$. **b** Particle filter ${\mathcal {O}} \big (\frac {N}{P}({log}_{2}N)^{3}\big)$

Figure 13 compares the performance of the original particle filtering algorithm when using the two variants of the redistribution component.

The comparative performance that was observed in the context of the redistribution component in isolation is also evident when comparing the performance of the overall particle filter. Indeed, the use of the ${\mathcal {O}}\left (\frac {N}{P}({log}_{2}N)^{2}\right)$ variant of redistribution results in (approximately) a fourfold increase in the number of particles processed per second. The trends that were observed in the context of the redistribution component in isolation are also apparent in the context of the overall particle filter.

### Speedup and scalability analysis

We now focus on the speedup that the ${\mathcal {O}} \left (\frac {N}{P}({log}_{2}N)^{2}\right)$ variant of the redistribution component offers relative to the ${\mathcal {O}}\left (\frac {N}{P}({log}_{2}N)^{3}\right)$ variant and the scalability of the ${\mathcal {O}}\left (\frac {N}{P}({log}_{2}N)^{2}\right)$ variant, i.e., the extent to which using more cores improves performance.

We quantify speedup as the ratio of the number of particles per second for a fixed number of particles and number of cores. We quantify scalability, in the context of a fixed number of particles^27^, as the ratio of the number of particles per second with *N* cores relative to the number of particles per second with a single core.

We compare performance in the context of both platforms for various different numbers of particles.

#### Redistribution component in isolation

**Fig. 14:**
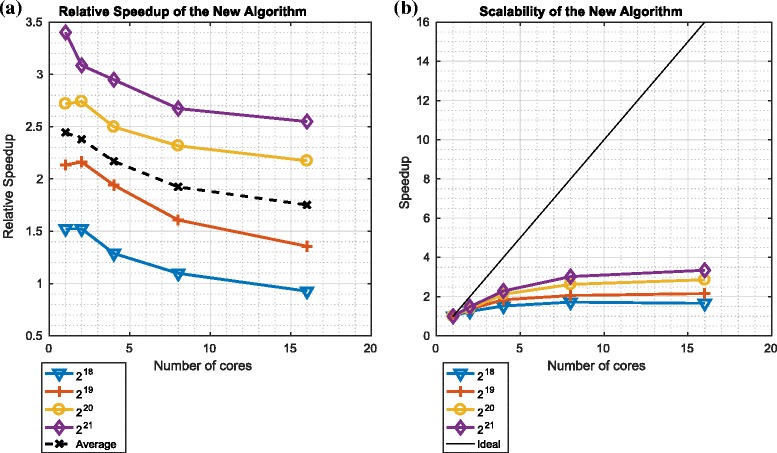
Relative speedup and scalability of the ${\mathcal {O}}\big (\frac {N}{P}({log}_{2}N)^{2}\big)$ variant of the redistribution component on platform 1. **a** Relative speedup: ${\mathcal {O}} \big (\frac {N}{P}({log}_{2}N)^{2}\big)$. **b** Scalability: ${\mathcal {O}} \big (\frac {N}{P}({log}_{2}N)^{2}\big)$

**Fig. 15:**
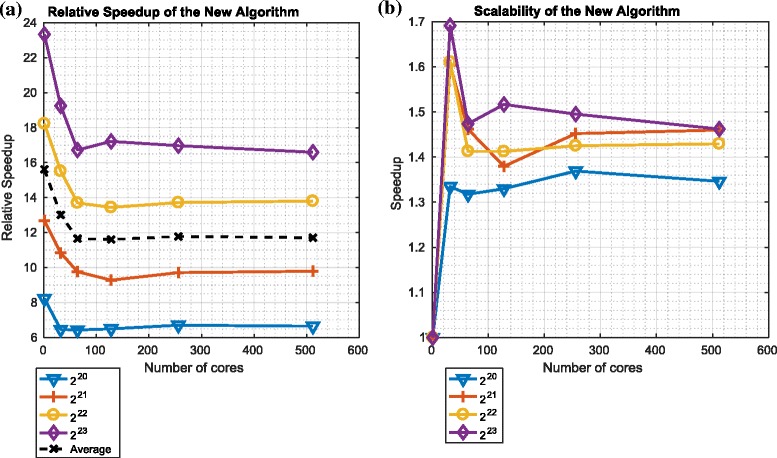
Relative speedup and scalability of the ${\mathcal {O}}\big (\frac {N}{P}({log}_{2}N)^{2}\big)$ variant of the redistribution component on platform 2. **a** Relative speedup: ${\mathcal {O}} \big (\frac {N}{P}({log}_{2}N)^{2}\big)$. **b** Scalability: ${\mathcal {O}} \big (\frac {N}{P}({log}_{2}N)^{2}\big)$

Figures 14 and 15 describe the speedup and scalability of the ${\mathcal {O}}\left (\frac {N}{P}({log}_{2}N)^{2}\right)$ redistribution component in the context of platforms 1 and 2 respectively.

We note that the relative speedup of the ${\mathcal {O}} \left (\frac {N}{P}({log}_{2}N)^{2}\right)$ variant of the redistribution component (relative to the ${\mathcal {O}}\left (\frac {N}{P}({log}_{2}N)^{3}\right)$ variant) is significant in all cases: between 2 (on platform 1) and 24 (on platform 2). For both platforms, this speedup increases as the number of particles is increased. However, we also note that, with platform 1 (which has a single node such that all cores share memory), the speedup decreases as the number of cores is increased for a fixed number of particles. In contrast, with platform 2, the speedup is broadly constant for large numbers of cores.

We also note that the scalability of the ${\mathcal {O}} \left (\frac {N}{P}({log}_{2}N)^{2}\right)$ variant of the redistribution component is far from ideal: increasing the number of cores culminates in minimal (if any) improvements in performance. This occurs because, in the context of both platforms, it is the communication, and not the computation, that is limiting performance. This also explains why platform 2’s larger number of cores does not offer improved scalability relative to platform 1: in platform 2, the processors are distributed across multiple nodes and communicate across a network, whereas platform 1’s processors are all part of the same node and so communicate using shared memory.

#### Resulting overall particle filter performance

**Fig. 16:**
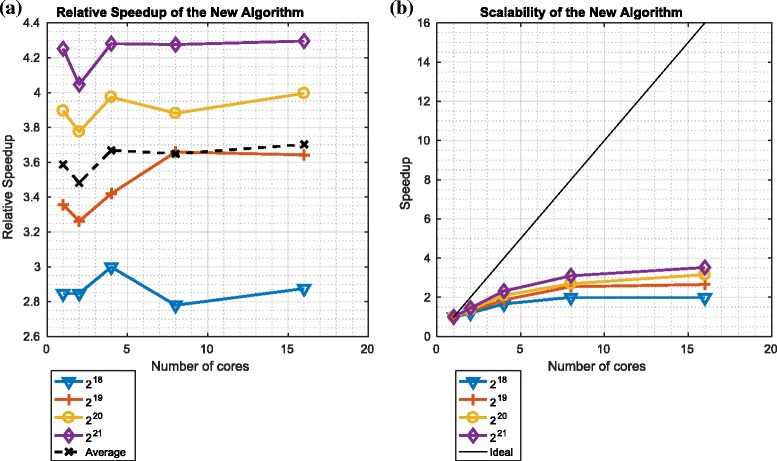
Relative speedup and scalability of the overall particle filter algorithm using the ${\mathcal {O}}\big (\frac {N}{P}({log}_{2}N)^{2}\big)$ variant of the redistribution component on platform 1. **a** Relative speedup: ${\mathcal {O}} \big (\frac {N}{P}({log}_{2}N)^{2}\big)$. **b** Scalability:${\mathcal {O}} \big (\frac {N}{P}({log}_{2}N)^{2}\big)$

**Fig. 17:**
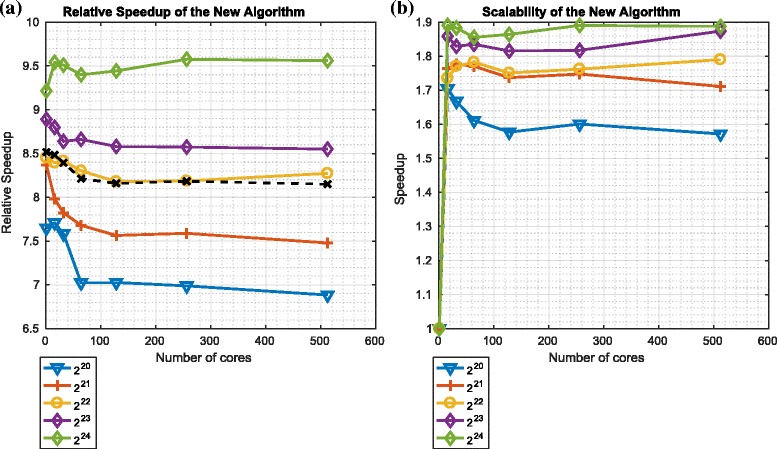
Relative speedup and scalability of the overall particle filter algorithm using the ${\mathcal {O}}\big (\frac {N}{P}({log}_{2}N)^{2}\big)$ variant of the redistribution component on platform 2. **a** Relative Speedup: ${\mathcal {O}} \big (\frac {N}{P}({log}_{2}N)^{2}\big)$. **b** Scalability: ${\mathcal {O}} \big (\frac {N}{P}({log}_{2}N)^{2}\big)$

Figures 16 and 17 describe the speedup and scalability of the overall particle filter using the ${\mathcal {O}}\left (\frac {N}{P}({log}_{2}N)^{2}\right)$ redistribution component in the context of platforms 1 and 2 respectively.

The speedups, as measured in the context of the overall particle filter algorithm are between 3 and 9.5. Again, for both platforms, the speedup increases with the number of particles. Again, the scalability is far from ideal.

### Discussion

The goal of the research is to dramatically reduce the execution time of particle filters. While we have gained significant insights from the performance analysis described herein (and hope that the paper will help others to also capitalise on those insights), the results described herein are ultimately disappointing: using the combination of algorithms and hardware considered herein, we are unable to improve on the execution speed achieved by a naïve redistribute implementation.

At one level, this is because the baseline against which we are comparing performance is relatively simple and mature. Our corresponding implementation is therefore relatively well optimised. In contrast, our proposed implementation is novel and has not been significantly optimised. However, we do not see it as fruitful to optimise our current implementation: we believe the disappointing results are caused by two other issues.

**Fig. 18:**
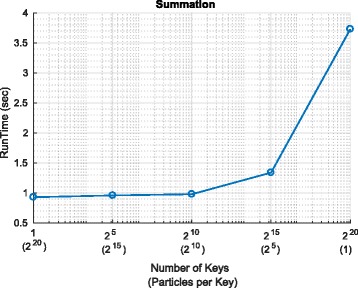
Performance of summation using Spark with a fixed total number of values comprised of different number of keys and therefore different numbers of values per key

The first issue is that, in our implementations, we have assumed that each particle has a unique key in the MapReduce framework. There are therefore as many keys as there are particles. To understand the potential benefit of having more than one value per key, we investigated how the performance of summation (in the context of a single core in platform 1 and 2^20^ values) changes as a function of the number of values per key. Figure 18 highlights that, for the example of summation in the context of a specific hardware configuration, a fourfold improvement in execution speed is possible by changing the number of values per key. This implies that runtime could change significantly if other components considered multiple particles to be associated with each key.

However, the primary issue which appears to be limiting runtime is the very MapReduce framework itself. As discussed in Section 2.2, before every Reduce operation, the values associated with each key are collated. This is a useful feature in the context of applications where the number of values associated with each key and the number of unique keys are unknown (e.g., where the task is to count up the number of occurrences of each word in a set of documents). However, in the particle filter considered herein, the number of unique keys is known to be the number of particles and the algorithms are chosen such that the number of values for each key are pre-defined for each algorithmic component. The flexibility that MapReduce provides is therefore of no utility in the context of the particle filter implementation. That lack of utility is not per se an issue. What is an issue is that the flexibility is achieved through a (somewhat hidden) ‘shuffle-and-sort’ phase that precedes every reduce operation. This phase (self-evidently from the name) in the versions or frameworks we used, was single-core bound. This sort is demanding in terms of communication and processing. So, every time MapReduce is used to perform even simple operations (e.g., cumulative sum), it is likely that the infrastructure is actually collating the keys and sorting them. Given the number of simple operations involved in our particle filter operation, we perceive it is this overhead that is dominating execution time.

This paper therefore motivates consideration of alternative frameworks which do not provision for the same flexibility as MapReduce and therefore do not have the same overheads. Our future work will therefore consider rethinking the entire manifestation of the implementation in alternative lower-level frameworks (e.g., using Message Passing Interface (MPI), a framework designed for HPC applications).

## Related work

A review of different resampling techniques is provided in [33]. This review makes clear that, at first sight, some of the key components of a particle filter, notably cumulative sum and redistribution, are inherently sequential^28^.

Indeed, this thinking has motivated research (e.g., as described in [44]) into approaches where a (small) number of processing elements (PEs) each perform local resampling and then communicate via a central process that, for example, allocates the particles to the PEs (a process that, as demonstrated in section 6, results in non-deterministic runtime). In contrast to the approaches involving communication between PEs, this paper is focused on a fully distributed algorithm (with no explicit central process and so no implicit assumption of a small number of PEs).

The detailed comparison of different (single-processor implementations of) resampling algorithms provided in [43] highlights that systematic resampling offers the best performance amongst the approaches considered. One strategy for parallel implementation (discussed in [44] and explored in more detail elsewhere [32]) is to deliberately choose an alternative resampling algorithm such that the alternative algorithm is more amenable to parallel implementation. This paper focuses on systematic resampling specifically.

Another approach that [33] highlights involves each particle performing resampling using only information from its local neighbours (e.g., as described in [34], which, in the view of the authors, does not make obvious that, if the resampling is performed locally then the weight after resampling should be proportional to the local normalising constant^29^). In contrast to approaches based on considering only local neighbours, this paper describes approaches that provide exactly the same output as a single processor would have generated.

Research not explicitly covered in the aformentioned review includes the implementation described in [9] and which this paper explicitly builds upon. That implementation achieves ${\mathcal {O}}\left (\frac {N}{P}(\log N)^{3}\right)$ time complexity with *N* parallel processors (and achieves a runtime that is not data dependent). Other related research includes (in [35]) a more complex, parallelised particle filter that uses a context-aware scheduling algorithm. They address the load imbalance arising from the naïve parallelisation of the particle filtering by using a custom (but reusable) scheduler. In this paper, we replace the use of such a scheduler at runtime by algorithmic development at design-time.

There has been previous work on implementing particle filters in a MapReduce context (e.g., in [7, 8]). However, this research has focused on using Hadoop and has not included a similar analysis to that documented in Section 6 of this paper. Our analysis in that section of this paper indicates that substantial improvements are possible using Spark but also highlights that the speedup offered using MapReduce and large numbers of processors is somewhat disappointing.

## Conclusions

In this paper, we have developed an improved parallel particle filtering algorithm. The core novelty is a novel redistribution component. The component provides deterministic runtime and a time complexity of ${\mathcal {O}}\left (\frac {N}{P}(\log N)^{2}\right)$ (with *N* particles and *N* processors). This improves on a previous approach that achieved a time complexity of ${\mathcal {O}} \left (\frac {N}{P}(\log N)^{3}\right)$.

A particle filter (including both the previous and new redistribution components) has been implemented using two Big Data frameworks, Hadoop and Spark. Rather than assuming that the performance of such an implementation will be faster than a single-core version, extensive performance evaluation has been conducted. Our new component outperforms the original version in isolation and when considering a particle filter that uses the new component in place of the original version. Our results indicate that, in the context of a particle filter, Spark’s ability to perform calculations in memory enable it to offer a 25-fold improvement in runtime relative to Hadoop. Using Spark and our new component, we go on to show that, as the number of particles increases, so does the implementation efficiency.

This performance evaluation highlights that it is not always valid to assume that porting algorithms to Big Data frameworks will result in an increase in execution speed. Indeed, the implementation we evaluated is limited by the communications overhead necessarily associated with giving each particle a unique key in the MapReduce framework: as a result, while we can achieve a speedup of threefold with 16 cores in a single node, with 512 cores spread across 28 nodes, we only achieve a speedup of approximately 1.4 (i.e., less). Furthermore, our implementation is outperformed by a naïve implementation by a factor of approximately 20. Put simply, using our current implementation, we cannot yet outperform an (optimised) single-processor resampling algorithm.

Of course, there will be applications where resampling is a small fraction of the total computational cost of the particle filter. In such contexts, the proposal, likelihood and/or dynamic model will be computationally demanding to calculate. These components of the particle filter are trivial to parallelise. Our future work will aim to broaden the applicability of our results beyond those applications. More specifically, we plan to focus on architectures involving a single key being related to multiple particles, explicitly minimising the need for data movement and removing the large lineages that appear to be limiting the performance possible using Spark.

Finally, we note that we have made our implementations available for public access via an OpenSource repository at GitHub as particlefilter [30].

## Endnotes

^1^ Though we are not aware of any empirical analysis of this approach being published.

^2^ More specifically, the algorithm’s runtime is independent of the distribution of the weights in the particle filter.

^3^ Including the associated ever-growing ecosystem of tools (e.g., Mahout [31] and GraphX for Spark [36]).

^4^ Conventional High Performance Computing (HPC) approaches use parallel computations to optimise processing time. We refer the reader to [37] for a good coverage of HPC-bound approaches for parallelising applications.

^5^ We anticipate that the ‘heat wall’ (i.e., the inability to remove enough heat from transistors that switch ever faster) will mean that for chip manufacturers to meet the expectation set by Moore’s law, they will soon (if not already) be doubling the number of cores (not transistors per square inch) used in each processor each year. In ten years’ time, if this trend continues, we would have desktop computers with a thousand times as many cores as today. This trend motivates the authors to design implementation strategies for particle filters that are well suited to the multi-core processors which will, we believe, become increasingly prevalent over time.

^6^ This contrasts HPC-based solutions, where the programmer aims to exploit intricate knowledge of the underlying architecture to ensure that data movement and processing are jointly optimised for the specific hardware.

^7^ The exact number of mapper and reducer processes on a parallel resource (for instance, a multi-node cluster) varies depending on the configuration, but the important point is that the algorithm developer does not need to worry about how the processes are distributed when defining the algorithm. Of course, that does not mean that there is not utility in the developer describing algorithms using mappers and reducers that are well suited to the problem being tackled and to the configuration being used.

^8^ Note that the output from each sentence would only be for the words that occur in that sentence, not every word that ever occurs in the corpus.

^9^ This can make it hard for a programmer to debug algorithmic implementations, particularly if the programmer is unfamiliar with debugging software performing lazy evaluation.

^10^ While the derivation involves consideration of a path, the resulting algorithm only needs to store the most recent state.

^11^ A good choice of proposal density can delay but not stop the effect [22].

^12^ This, of course, leads to a loss of diversity among the particles.

^13^ For instance, although Quicksort [28] can be parallelised, the load distributions across the processors is dependent on the pivots used and the run-time will therefore be data-dependent.

^14^ Such operations are an example of ‘embarrassingly parallel’ operations that are arguably trivial to parallelise.

^15^ Note that the cumulative sum is sometimes referred to as a prefix sum: there is no difference between a prefix sum and a cumulative sum.

^16^ APL describes an approach to calculating a sum, maximum or minimum as *reduction* operations. The approach to calculating a cumulative sum is described as a *scan* operation and can be used to calculate, for example, cumulative maximums and minimums. Scan operations take a binary operator ⊕ and an *N*-element vector **a**=[*a*_1_,*a*_2_,…,*a*_*N*_], and return an *N*-element vector **a**_⊕_=[*a*_1_,(*a*_1_⊕*a*_2_),…,(*a*_1_⊕*a*_2_⊕*a*_3_⊕…⊕*a*_*N*_)]. However, here we are only concerned with cumulative sums.

^17^ The ceiling of *x* is the smallest integer larger than or equal to *x*.

^18^ The floor of *x* is the largest integer smaller than or equal to *x*.

^19^ A similar argument works if the first half are sorted in descending order and the second half are sorted in ascending order.

^20^ This process is sometimes known as ‘bitonic build’.

^21^ The first dataset is actually already sorted, but the second dataset is, in general, not sorted.

^22^ We suspect such a sequence may have a name in a literature we do not currently have sight of. However, here we simply adopt an intuitive name for ease of exposition.

^23^ In subsequent sections, we will investigate how and when the decrease in runtime occurs in more detail.

^24^ With *N* particles, the best-case involves replicating each particle exactly once.

^25^ Since Hadoop does not attempt lazy evaluation or use such lineages for another purpose, the same phenomenon is not observed in the context of Hadoop.

^26^ In the particle filter the resampling is executed in every iteration. Thus the aforementioned figures correspond to a worst-case speedup.

^27^ Since the problem size remains fixed, we are actually quantifying *strong scaling* [38].

^28^ The review also highlights challenges associated with, for example, multiple processors generating independent random number sequences, discusses the relative merits of using floating-point and fixed-point numbers and points to papers discussing architecture-specific issues (e.g., in [39–42]).

^29^ More mathematically, assume the *i*th particle has a weight (before resampling) of *w*_*i*_ and the *j*th member of the new population is resampled as a copy of the *i*th particle with probability of $\frac {w_{i}}{\sum _{i'\in I_{j}}w_{i'}}$ where *I*_*j*_ is the set of particles that are local to the *j*th particle. The (unnormalised) weight after resampling (based on considering the resampling process in terms of importance sampling) is $w_{i}\times \frac {\sum _{i'\in I_{j}}{w_{i'}}}{w_{i}}=\sum _{i'\in I_{j}}{w_{i'}}$. The normalised weight would then be proportional to this unnormalised weight, but scaled such that the normalised weight sums to one over all particles.

## References

[1] Gordon NJ, Salmond DJ, Smith AFM (1993). Novel approach to nonlinear/non-Gaussian Bayesian state estimation. IEE Proc. F - Radar Signal Process.

[2] Creal D (2012). A survey of sequential Monte Carlo methods for economics and finance. Econ. Rev.

[3] Gustafsson F, Gunnarsson F, Bergman N, Forssell U, Jansson J, Karlsson R, Nordlund PJ (2002). Particle filters for positioning, navigation, and tracking. IEEE Trans. Signal Process.

[4] T Sakaki, M Okazaki, Y Matsuo, in *Proceedings of the 19th International Conference on World Wide Web*. Earthquake shakes Twitter users: real-time event detection by social sensors (ACM, 2010), pp. 851–860. https://dl.acm.org/citation.cfm?id=1772777.

[5] Thrun S, Fox D, Burgard W, Dellaert F (2001). Robust Monte Carlo localization for mobile robots. Artif. Intell.

[6] Liu JS (1996). Metropolized independent sampling with comparisons to rejection sampling and importance sampling. Stat. Comput.

[7] Bai F, Gu F, Hu X, Guo S (2016). Particle routing in distributed particle filters for large-scale spatial temporal systems. IEEE Trans. Parallel Distrib. Syst.

[8] F Bai, X Hu, in *Proceedings of the High Performance Computing Symposium, HPC ’13*. Cloud MapReduce for particle filter-based data assimilation for wildfire spread simulation (Society for Computer Simulation International, 2013), pp. 1–6. https://dl.acm.org/citation.cfm?id=2499979.

[9] S Maskell, B Alun Jones, M Macleod, in *Nonlinear Statistical Signal Processing Workshop*. A Single instruction multiple data particle filter (IEEE, 2006), pp. 51–54. http://ieeexplore.ieee.org/document/4378818/.

[10] Apache Hadoop (2016). http://hadoop.apache.org. Accessed 29 Mar 2017.

[11] Apache Spark (2016). http://spark.apache.org. Accessed 29 Mar 2017.

[12] Apache Storm (2016). http://storm.apache.org. Accessed 29 Mar 2017.

[13] M Schroeck, R Shockley, J Smart, D Romero-Morales, P Tufano, Analytics: the real-world use of Big data (2012). IBM Institute for Business Value, IBM Institute for Business Value - Executive Report.

[14] Reyes-Ortiz JL, Oneto L, Anguita D (2015). Big Data analytics in the Cloud: Spark on Hadoop vs MPI/OpenMP on Beowulf. Procedia Comput. Sci.

[15] Dean J, Ghemawat S (2008). MapReduce: simplified data processing on large clusters. Commun. ACM.

[16] C Herath, B Plale, in *CCGRID*. Streamflow programming model for data streaming in scientific workflows (IEEE Computer Society, 2010), pp. 302–311. http://ieeexplore.ieee.org/document/5493467/?reload=true.

[17] Apache Pig and Latin (2016). http://pig.apache.org. Accessed 29 Mar 2017.

[18] Wang H, Qin X, Zhou X, Li F, Qin Z, Zhu Q, Wang S (2015). Efficient query processing framework for Big data warehouse: an almost join-free approach. Front. Comput. Sci.

[19] Zaharia M, Chowdhury M, Das T, Dave A, Ma J, McCauly M, Franklin MJ, Shenker S, Stoica I (2012). Resilient distributed datasets: a fault-tolerant abstraction for in-memory cluster computing. Ninth USENIX Symposium on Networked Systems Design and Implementation (NSDI 12).

[20] Arulampalam MS, Maskell S, Gordon N, Clapp T (2002). A tutorial on particle filters for online nonlinear/non-Gaussian Bayesian tracking. IEEE Trans. Signal Process..

[21] JM Hammersley, DC Handscomb, *Monte Carlo Methods* (Wiley, 1964). https://link.springer.com/book/10.1007%2F978-94-009-5819-7.

[22] Doucet A, Godsill S, Andrieu C (2000). On sequential monte carlo sampling methods for Bayesian filtering. Stat. Comput.

[23] Kong A, Liu JS, Wong WH (1994). Sequential imputations and Bayesian missing data problems. J. Am. Stat. Assoc.

[24] Isard M, Blake A (1998). CONDENSATION—conditional density propagation for visual tracking. Int. J. Comput. Vis.

[25] Kitagawa G (1996). Monte Carlo filter and smoother for non-Gaussian nonlinear state space models. J. Comput. Graph. Stat.

[26] GE Blelloch, Prefix sums and their applications (1990). Technical Report CMU-CS-90-190, School of Computer Science, Carnegie Mellon University.

[27] Iverson KE (1962). A Programming Language.

[28] Hoare CAR (1961). Algorithm 64: Quicksort. Commun. ACM.

[29] KE Batcher, in *Proceedings of the Spring Joint Computer Conference, AFIPS ’68 (Spring)*. Sorting networks and their applications (ACM, 1968), pp. 307–314. https://dl.acm.org/citation.cfm?id=1468121.

[30] Particle Filter Repository (2017). https://github.com/particlefilter/mrpf. Accessed 29 Mar 2017.

[31] Apache Mahout (2016). http://mahout.apache.org. Accessed 29 Mar 2017.

[32] Murray LM, Lee A, Jacob PE (2016). Parallel resampling in the particle filter. J. Comput. Graph. Stat.

[33] Li T, Bolic M, Djuric PM (2015). Resampling methods for particle filtering: classification, implementation, and strategies. IEEE Signal Process. Mag.

[34] Míguez J, Bugallo MF, Djurić PM (2004). A new class of particle filters for random dynamic systems with unknown statistics. EURASIP J. Adv. Signal Process.

[35] Sutharsan S, Kirubarajan T, Lang T, Mcdonald M (2012). An optimization-based parallel particle filter for multitarget tracking. IEEE Trans. Aerospace Electronic Syst.

[36] Singh D, Reddy CK (2014). A survey on platforms for big data analytics. J. Big Data.

[37] Asanovic K, Bodik R, Demmel J, Keaveny T, Keutzer K, Kubiatowicz J, Morgan N, Patterson D, Sen K, Wawrzynek J, Wessel D, Yelick K (2009). A view of the parallel computing landscape. Commun. ACM.

[38] Hill MD (1990). What is scalability?. SIGARCH Comp. Arch. News.

[39] G Hendeby, JD Hol, R Karlsson, F Gustafsson, in *Proceedings of the 15th European Signal Processing Conference*. A graphics processing unit implementation of the particle filter (IEEE, 2007), pp. 1639–1643. http://ieeexplore.ieee.org/document/7099084/.

[40] Hendeby G, Karlsson R, Gustafsson F (2010). Particle filtering: the need for speed. EURASIP J. Adv. Signal Process.

[41] Hong S, Chin SS, Djurić PM, Bolić M (2006). Design and implementation of flexible resampling mechanism for high-speed parallel particle filters. J. VLSI Signal Process. Syst. Signal Image Video Technol.

[42] Hwang K, Sung W (2013). Load balanced resampling for real-time particle filtering on graphics processing units. Transa. Signal Process.

[43] JD Hol, TB Schon, F Gustafsson, in *Nonlinear Statistical Signal Processing Workshop*. On resampling algorithms for particle filters (IEEE, 2006), pp. 79–82. http://ieeexplore.ieee.org/document/4378824/.

[44] Bolic M, Djuric PM, Hong S (2005). Resampling algorithms and architectures for distributed particle filters. IEEE Trans. Signal Process.

